# PKCθ and HIV-1 Transcriptional Regulator Tat Co-exist at the LTR Promoter in CD4^+^ T Cells

**DOI:** 10.3389/fimmu.2016.00069

**Published:** 2016-02-29

**Authors:** María Rosa López-Huertas, Jasmine Li, Anjum Zafar, Sara Rodríguez-Mora, Carlota García-Domínguez, Elena Mateos, José Alcamí, Sudha Rao, Mayte Coiras

**Affiliations:** ^1^AIDS Immunopathology Unit, National Center of Microbiology, Instituto de Salud Carlos III, Madrid, Spain; ^2^Department of Microbiology and Immunology, The Doherty Institute for Infection and Immunity, University of Melbourne, Melbourne, VIC, Australia; ^3^Biomedical Sciences, Faculty of Education, Science, Technology and Mathematics, University of Canberra, Canberra, ACT, Australia; ^4^Functional Research Unit in Chronic Diseases, National Center of Microbiology, Instituto de Salud Carlos III, Madrid, Spain

**Keywords:** HIV-1 Tat regulator, protein kinase C theta, Ras/Raf/MEK/Erk pathway, NF-kappa B, nuclear colocalization, CD4+ T cell activation, PKC theta mRNA interference, PKC theta and HIV-1 Tat interaction

## Abstract

PKCθ is essential for the activation of CD4^+^ T cells. Upon TCR/CD28 stimulation, PKCθ is phosphorylated and migrates to the immunological synapse, inducing the activation of cellular transcription factors such as NF-κB and kinases as ERK that are critical for HIV-1 replication. We previously demonstrated that PKCθ is also necessary for HIV-1 replication but the precise mechanism is unknown. Efficient HIV-1 transcription and elongation are absolutely dependent on the synergy between NF-κB and the viral regulator Tat. Tat exerts its function by binding a RNA stem-loop structure proximal to the viral mRNA cap site termed TAR. Besides, due to its effect on cellular metabolic pathways, Tat causes profound changes in infected CD4^+^ T cells such as the activation of NF-κB and ERK. We hypothesized that the aberrant upregulation of Tat-mediated activation of NF-κB and ERK occurred through PKCθ signaling. In fact, Jurkat TetOff cells with stable and doxycycline-repressible expression of Tat (Jurkat-Tat) expressed high levels of mRNA for PKCθ. In these cells, PKCθ located at the plasma membrane was phosphorylated at T^538^ residue in undivided cells, in the absence of stimulation. Treatment with doxycycline inhibited PKCθ phosphorylation in Jurkat-Tat, suggesting that Tat expression was directly related to the activation of PKCθ. Both NF-κB and Ras/Raf/MEK/ERK signaling pathway were significantly activated in Jurkat-Tat cells, and this correlated with high transactivation of HIV-1 LTR promoter. RNA interference for PKCθ inhibited NF-κB and ERK activity, as well as LTR-mediated transactivation even in the presence of Tat. In addition to Tat-mediated activation of PKCθ in the cytosol, we demonstrated by sequential ChIP that Tat and PKCθ coexisted in the same complex bound at the HIV-1 LTR promoter, specifically at the region containing TAR loop. In conclusion, PKCθ-Tat interaction seemed to be essential for HIV-1 replication in CD4^+^ T cells and could be used as a therapeutic target.

## Introduction

The human immunodeficiency virus type 1 (HIV-1) infection is characterized by a continuous viral replication in CD4^+^ T lymphocytes and macrophages (1), ultimately leading to the development of the acquired immunodeficiency syndrome (AIDS). The preferential targets of HIV-1 are the activated CD4^+^ T cells from the gut-associated lymphoid tissue (GALT) (2, 3), where the virus performs a massive replication during the early stages of the illness. During this massive activation, two major events occur. First, viral reservoirs with the provirus integrated in the host-cell DNA are generated and persist in latency, representing an insuperable obstacle for the eradication of the infection (4–6). Second, the major target of HIV-1, CD4^+^ T cells, is progressively depleted, which eventually leads to AIDS (7, 8). Although HIV-1 may infect both resting and activated CD4^+^ T cells, it can only replicate in activated cells (9). Therefore, CD4^+^ T cell activation is essential to sustain HIV-1 replication.

The novel protein serine/threonine kinase C (PKC) theta (θ) isoform is selectively expressed in T lymphocytes (10) and is essential for TCR/CD3-mediated T-cell activation (11, 12). PKCθ kinase activity is regulated by phosphorylation of different residues during T-cell activation (13, 14). Phosphorylation of T^538^ on the catalytic domain has been described as critical for PKCθ kinase activity and for inducing NF-κB activation (15). Therefore, the phosphorylation of this residue is often used as surrogate marker of PKCθ activation (14) and can be detected in dividing cells. Once phosphorylated at T^538^, PKCθ is translocated to the plasma membrane lipid rafts during the immunological synapse (16), inducing the activation of signaling pathways such as Ras/Raf/MEK/ERK and transcription factors such as NF-κB (17). NF-κB is critical for the regulation of key immune genes as interleukin-2 (IL-2) (12, 18) and for the replication of HIV-1 (19). Our group previously described that PKCθ catalytic activity is essential for HIV-1 replication, as shown by PKCθ mRNA interference (20) or the use of selective PKCθ inhibitors (21), which reduced HIV-1 replication in CD4^+^ T cells and induced a refractory state to HIV-1 infection. Although the role of PKCθ in the cytoplasm and at the immunological synapse has been extensively studied, PKCθ was recently described in the nucleus of activated T cells as part of an active transcription complex with RNA polymerase II (RNAPII) assembled at the *IL-2* promoter (22).

The viral transcriptional regulator Tat is critical for HIV-1 gene expression and replication (23). Tat binding site within the viral 5′ long terminal repeat (LTR) promoter of the integrated provirus is a stem-loop RNA termed trans-activation response (TAR) element that is located at the 5′-end of all nascent viral transcripts (24). TAR is located downstream the viral LTR promoter, a region spanning from nucleotide position +1 to +59. Tat/TAR binding enables efficient viral transcript elongation through the recruitment of cellular factors from the basal transcriptional machinery such as the positive transcription elongation factor b (P-TEFb) to increase the processivity of RNAPII (25). P-TEFb is composed of cyclin T1, which directly interacts with Tat to permit the binding to TAR (26), and the cyclin-dependent kinase 9 (CDK9) that hyperphosphorylates RNAPII at the carboxy terminal domain (CTD) to ensure efficient elongation of the viral transcripts (27). Tat shows a predominant nuclear distribution in infected CD4^+^ T cells although it can also be released into the extracellular medium and taken up by adjacent non-infected cells (28). The *tat* coding gene consists of two spliced exons separated by more than 2,300 nucleotides in the HIV-1 genome. After complete splicing of the viral pre-mRNA, a highly conserved protein composed of 101 residues is synthesized (Tat101) (29). The first exon of Tat (1–72 aa, Tat72) contains the minimal functional domain to generate a protein competent in HIV-1 replication (30) but Tat101 is the most common protein in clinical HIV-1 isolates (31). Our group previously demonstrated that Tat101 was more competent than Tat72 in causing deregulation in gene expression and cytoskeleton modifications due to the presence of the peptide encoded by the second exon, a high-positive charged region of 29 aa that would enhance the binding strength or affinity to host cell targets, providing more activity to Tat101 than Tat72 (32). As part of the modifications caused in the CD4^+^ T cells, Tat induces the activation of several transcription factors in CD4^+^ T cells such as NF-κB, NFAT, Sp1, and kinases such as ERK (32–34), although the precise mechanisms involved are currently controversial.

In this study, we analyzed the effect of two essential factors, cellular PKCθ and viral Tat, on HIV-1 replication and their possible interaction in the nucleus of CD4^+^ T cells. PKCθ-mediated pathways such as Ras/Raf/MEK/ERK and NF-κB were analyzed in Jurkat cells with stable expression of Tat101 and Tat72 proteins. Even in the absence of stimulation, Jurkat-Tat101 cells showed increased PKCθ expression levels as well as T^538^ phosphorylation, which correlated with increased Ras/Raf/MEK/ERK and NF-κB activity. Analysis of nuclear colocalization and chromatin interaction provided support for the coexistence of PKCθ and Tat within the nucleus, and that they were interacting at the LTR promoter, specifically within the region of TAR loop.

## Materials and Methods

### Cells

Jurkat E6-1 cells were obtained from the NIH AIDS Reagent Program (35). By using a TetOff system (BD Biosciences Clontech, Mountain View, CA, USA), Jurkat-Tat72 and Jurkat-Tat101 stably express the first exon of HIV-1 Tat (1–72 aa) or the full-length Tat (1–101 aa), respectively. Jurkat TetOff cells transfected with empty vector pTRE2hyg were used as negative control. Jurkat-Tat72 and Jurkat-Tat101 are not clones but mixed populations in which more than 50% of the cells express high amounts of intracellular Tat101 or Tat72 protein. The expression of Tat in Jurkat-Tat101 and Jurkat-Tat72 mimicked the real infection performed in MT-2 cells infected with the NL4.3wt strain (32). Jurkat E6-1 cells were cultured in RPMI 1640 medium supplemented with 10% (*v*/*v*) fetal calf serum (FCS), 2 mM l-glutamine, 100 μg/ml streptomycin, and 100 U/ml penicillin (Biowhittaker, Walkersville, MD, USA). In Jurkat-Tat cells, the culture medium was supplemented with 300 μg/ml of geneticin (Sigma-Aldrich, St. Louis, MO, USA) and 300 mg/ml hygromycin B (BD Biosciences Clontech). Tat expression was controlled by adding 1 μg/ml doxycycline (BD Biosciences Clontech) to the culture medium for 3 days. Peripheral blood lymphocytes (PBLs) were isolated from blood of healthy donors by centrifugation through a Ficoll–Hypaque gradient (Lymphocyte Separation Medium, Lonza). Cells were collected in supplemented RPMI 1640 medium and maintained at 37°C, 2 × 10^6^ cells/ml. Phytohemagglutinin (PHA)-treated T lymphocytes were obtained from PBLs cultured for 3 days in the presence of 5 μg/ml PHA (Sigma-Aldrich, St. Louis, MO, USA) and 300 U/ml IL-2 (Chiron, Emeryville, CA, USA).

### Vectors

Plasmid pCMV-tat101 was previously described (36). Long terminal repeat (LTR) vector containing the luciferase (LUC) reporter gene under the control of HIV-1 LTR U3 + R region (LAI strain) (pLTR-LUC) was described previously (37). To detect Elk-1 activity, Jurkat cells were cotransfected with 16 ng pCDNAIII-Gal4-Elk1, 0.1 μg pRL-TK (containing the Renilla LUC gene under control of the HSV-TK promoter), and 0.3 μg pGal4-LUC (containing *luc* gene controlled by six copies of Gal4 responsive element) per million of cells, as previously described (38). Vector pNL4-3 wild-type (wt) kindly provided by Dr. M. A. Martin (39) contained the HIV-1 complete genome and induced an infectious progeny after transfection. pNL4-3_TatM1I, which contains a point mutation in the start codon of the *tat* gene, was obtained from pNL4-3_wt by site-directed mutagenesis as previously described (40). pNL4-3_TatM1I was cotransfected along with pCMV-Tat101 in a 2:1 proportion. GeneClip U1 Hairpin Cloning System kit (Promega Biotech Iberica, Madrid, Spain) was used to generate the small hairpin RNA (shRNA) plasmids pGeneClip-iPKCθ-1 and pGeneClip-iPKCθ-3 containing two different small interference RNA (siRNA) sequences directed against mRNA encoding for PKCθ, and the control vector pGeneClip-iPKCθ-C1 with scrambled sequences, as was described in Ref. (40). All plasmids were purified using Qiagen Plasmid Maxi Kit (Qiagen Iberia, Madrid, Spain), following the manufacturer’s instructions.

### Antibodies and Reactives

Monoclonal antibody against HIV-1 Tat (aa 2–9) was obtained from Advanced Biotechnologies Inc. (Columbia, MD, USA). Specific antibody against p65/RelA (clone C-20) was supplied by Santa Cruz Biotechnology (Santa Cruz, CA, USA). Specific antibody against the β-isoform of actin was obtained from Sigma Aldrich. Polyclonal antibodies against human PKCθ and PKCδ were purchased from Santa Cruz Biotechnology. Polyclonal antibodies against phospho-PKCθ (Thr^538^), phospho-PKCδ (Thr^505^), total c-Raf/Raf-1, phospho-c-Raf/Raf-1 (Ser^338^), total MEK1/2, phospho-MEK1/2 (Ser^217/221^), total p44/42 MAPK (Erk1/2), and phospho-Erk1/2 (Thr^202^/Tyr^204^) were obtained from Cell Signaling Technology (Danvers, MA, USA). Secondary antibodies conjugated with horseradish peroxidase (HRP) were purchased from GE Healthcare (Uppsala, Sweden). Secondary antibodies conjugated to Alexa 488, Alexa 555, and Alexa 633 were purchased from Molecular Probes (Eugene, OR, USA). 5-phorbol 12-myristate 13-acetate (PMA) (Sigma-Aldrich) was used at 25 ng/ml.

### Quantitative PCR Assay

Total RNA was isolated with RNeasy Mini kit (Qiagen Iberia), and cDNA was synthesized by using the GoScript Reverse Transcription System (Promega), according to manufacturers’ instructions. HIV-1 transcription was determined by quantitative PCR (qPCR) analysis of all viral mRNAs using the following primers to amplify *PRKCQ* gene: PKCt7_s (5′-GCAATTTCGACAAAGAATT-3′) and PKCt5_as (5′-ATTGTTGAGTGTTTCTTTC-3′); and *PRKCD* gene: PKCd3_s (5′-GCTGCCATCCACAAGAAAT-3′) and PKCd4_as (5′-ACTTTAATCCCTGCTTCAC-3′). The expression of *β-actin* was used as housekeeping gene to calculate the relative expression of *PRKCQ* and *PRKCD*. Primers for amplifying human *β-actin* gene were β-actin-s (5′-AGGCCCAGAGCAAGAGAGGCA-3′) and β-actin-as (5′-CGCAGCTCATTGTAGAAGGTGTGGT-3′). qPCR was performed in a StepOnePlus Real-Time PCR System, using StepOne v3.2 software (Life Technologies, Carlsbad, CA, USA). SYBR Green PCR Master Mix (Applied Biosystems) was used according to manufacturer’s instructions.

### PKC Kinase Assays

PKCθ and PKCδ enzymatic activity was assayed in Jurkat-Tat cells using an immunoprecipitation kinase assay as described previously (41), with minor modifications as described in Ref. (20). Protein extracts (250–500 μg) were immunoprecipitated for 18 h at 4°C using 2 μg of anti-PKCθ or anti-PKCδ antibodies. The immunoprecipitates were incubated with goat anti-rabbit IgG (whole molecule) antibody conjugated with agarose (Sigma-Aldrich). The enzymatic reaction was performed by using PKCϵ peptide substrate ERMRPRKRQGSVRRRV (Merck Millipore, Darmstadt, Germany). As a negative control, one reaction with the goat anti-rabbit IgG antibody conjugated with agarose (Sigma-Aldrich) was performed without adding the primary antibody to the cytosolic extracts. A complete reaction mixture lacking of PKCϵ peptide substrate was used as blank solution. Radioactivity was counted in a Beckman LS 6000L scintillation counter (Beckman Instruments, Fullerton, CA, USA). The corrected cpm (counts per minute) was determined by subtracting the blank control value.

### Transient Transfection

Jurkat cells were transiently transfected with an Easyjet Plus Electroporator (Equibio, Middlesex, UK). In brief, 20 × 10^6^ PBLs or 10^7^ Jurkat were collected in 350 μl of RPMI without supplement and mixed with 1 μg/10^6^ cells of each plasmid DNA. Cells were transfected in a cuvette with 4-mm electrode gap (EquiBio) at 280 V, 1500 μF, and maximum resistance. After transfection, cells were incubated in supplemental RPMI at 37°C for 18 h. LUC activity was assayed using Luciferase Assay System (Promega), according to manufacturer’s instructions. Relative light units (RLUs) were measured with a Sirius luminometer (Berthold Detection Systems, Oak Ridge, TN, USA) after the addition of the appropriate substrate. RLUs were normalized by measuring total protein concentration with the method of Bradford (42). pEYFP-C1 vector (BD Biosciences Clontech) was cotransfected as control of transfection efficiency and measured by flow cytometry using a FACScalibur Flow Cytometer and CellQuest software (BD Biosciences). Interference of mRNA for PKCθ was performed by transient transfection of Jurkat cells with pGeneClip-iPKCθ-1 and pGeneClip-iPKCθ-3 vectors (1:1), using pGeneClip-iPKCθ-C1 as control.

### HIV-1 Infection

Briefly, 10 × 10^6^ of Jurkat E6-1 cells were incubated 2 h at gently rotation and room temperature with 0.5 μg/ml of p24-gag of NL4-3 infectious supernatant obtained from calcium phosphate transfection of 293T cells with plasmid pNL4-3_wt. Cells were then centrifuged at 2,500 rpm for 30 min at 25°C, and washed five times with 1× PBS. After incubation for 7 days, cells were analyzed by immunofluorescence. Quantification of p24 was performed by Innotest HIV Antigen mAb assay (Innogenetics Diagnostica Iberia, Barcelona, Spain).

### Immunofluorescence Assays

For immunofluorescence assays, cells were immobilized in PolyPrep slides (Sigma-Aldrich) for 15 min and then fixed with 2% paraformaldehyde (PFA)–0.025% glutaraldehyde in 1× PBS for 10 min at room temperature. After washing twice with 0.1% glycine/PBS, cells were permeabilized with 0.1% Triton X-100/PBS. Incubation with each primary and secondary antibodies and subsequent washes were performed with 1× PBS–2% BSA–0.05% saponine buffer. 4′,6-diamidino-2-phenylindole (Dapi) was used for nuclear staining while cholera toxin B subunit FITC (fluorescein isothiocyanate) conjugate was used as marker of membrane lipid rafts (Sigma-Aldrich). Images were obtained with Leica TCS-SP confocal microscope or Leica DMI 4000B Inverted Microscope (Leica Microsystems, Wetzlar, Germany). The intensity mean per pixel was calculated in confocal images using LAS AF Lite software (Leica Microsystems), and values were represented in bar diagrams showing statistical significance.

### Flow Cytometry

Intracellular staining was performed after fixing the cells with 1% PFA in 1× PBS for 1 h. Incubation with primary and secondary antibodies was performed with 1× PBS-0.5% Tween-20. Cells were analyzed on a FACScalibur Flow Cytometer using CellQuest software (BD Biosciences).

### Immunoblotting Assays

Cytosolic, nuclear, and membrane protein fractions were obtained as described previously (43, 44) and protein concentration was determined by Bradford method (42). Proteins were fractionated by sodium dodecyl sulfate-polyacrilamide gel electrophoresis (SDS-PAGE) and transferred onto Hybond-ECL nitrocellulose paper (GE Healthcare-Madrid, España). After blocking and incubation with primary and secondary antibodies, proteins were detected with SuperSignal West Pico Chemiluminescent Substrate (Pierce, Rockford, IL, USA). Images were acquired in a BioRad Geldoc 2000 (BioRad, Hercules, CA, USA), and densitometry analysis was performed using Quantity One software (BioRad). Relative ratio of optical density units was calculated regarding to the gel band corresponding to the internal control for each lane and each type of protein extracts after subtracting the background noise.

### DNA Affinity Immunoblotting Assay

DNA affinity immunoblotting (DAI) assay was performed as previously described in Ref. (45) with minor modifications. Briefly, 100 μg of nuclear protein extracts from each cell line were incubated at 4°C for 30 min with 25 nM of a 5′ end-labeled biotin probe that contains the double-κB consensus motif located in the HIV-1 LTR promoter (5′-AGCTTACAAGGGACTTTCCGCTG GGGACTTTCCAGGGA-3′), with this probe mutated at the -κB consensus sites (5′-AGCTTACAA*CTC*ACTTTCCGCTG*CTC* ACTTTCCAGGGA) or with a probe that contains the estrogen receptor (ER) DNA element, a non-related NF-κB gene (46). Biotin-probe/protein complexes were captured by incubating with streptavidin agarose resins (Pierce) at 4°C for 30 min and then collected by centrifugation. Protein complexes were fractionated by SDS-PAGE, and the presence of p65/RelA protein bound to DNA probes was detected by immunoblotting with a specific antibody (clone C-20; Santa Cruz Biotechnology). As internal control 10 μg of protein were separated before adding each probe and resolved by Western-blot using an antibody against β-actin (Sigma-Aldrich). Images were acquired in a BioRad Geldoc 2000 (BioRad), and densitometry analysis was performed using Quantity One software (BioRad) as described above.

### Assay for Detection of Activated Ras

Detection assay for activated Ras were performed as previously described (47) with modifications. Briefly, Jurkat-Tat cells were subjected to serum deprivation for 2 h and then lysed with lysis buffer (25 mM pH 7.5 HEPES, 1% Triton X-100, 150 mM NaCl, 10 mM MgCl_2_, and protease inhibitors). For affinity precipitation, lysates were incubated with purified GST-Raf-RBD protein pre-bound to glutation-Sepharose (GE Healthcare). Bound proteins were eluted and analyzed by immunoblotting using a specific antibody against N-Ras (SantaCruz Biotechnology). β-actin was used as internal loading control.

### Sequential Immunoprecipitation of Chromatin

Sequential immunoprecipitation of chromatin (ChIP) assays were performed on Jurkat E6-1 cells transfected with pNL4-3_wt according to the protocol supplied by Upstate Biotechnology, as previously detailed (22). Antibodies used were directed against PKCθ (clone C-18, SantaCruz Biotechnology) and HIV-1 Tat (clone 02-010, SantaCruz Biotechnology). qPCR with primers spanning across the region from −39 to +66 on the HIV-1 LTR promoter assessed the recovery of immunoprecipitated DNA, calculated as relative ChIP enrichment. The primers used were: sense 5′-TCAGATGCTACATATAAGCAGCTGCT-3′ and antisense 5′-AAGCAGTGGGTTCCCTAGTTAGCC-3′. ChIP-PCR data were collated from two independently repeated experiments (*N* = 2).

### Statistical Analysis

Statistical analysis was performed using GraphPad Prism software (GraphPad, La Jolla, CA, USA). Comparisons between groups were made using two-way analysis of variance (ANOVA) with Bonferroni posttest analysis to describe the statistical differences among groups. For comparison between less than three groups one-tailed Mann–Whitney test was used. The *P* values (*p*) < 0.05 were considered statistically significant in all comparisons and were represented as *, **, ***, or **** for *p* < 0.05, *p* < 0.01, *p* < 0.001, or *p* < 0.0001, respectively.

## Results

### Infection of Jurkat Cells with HIV-1 Induced PKCθ Phosphorylation at T^538^

**Figure 1:**
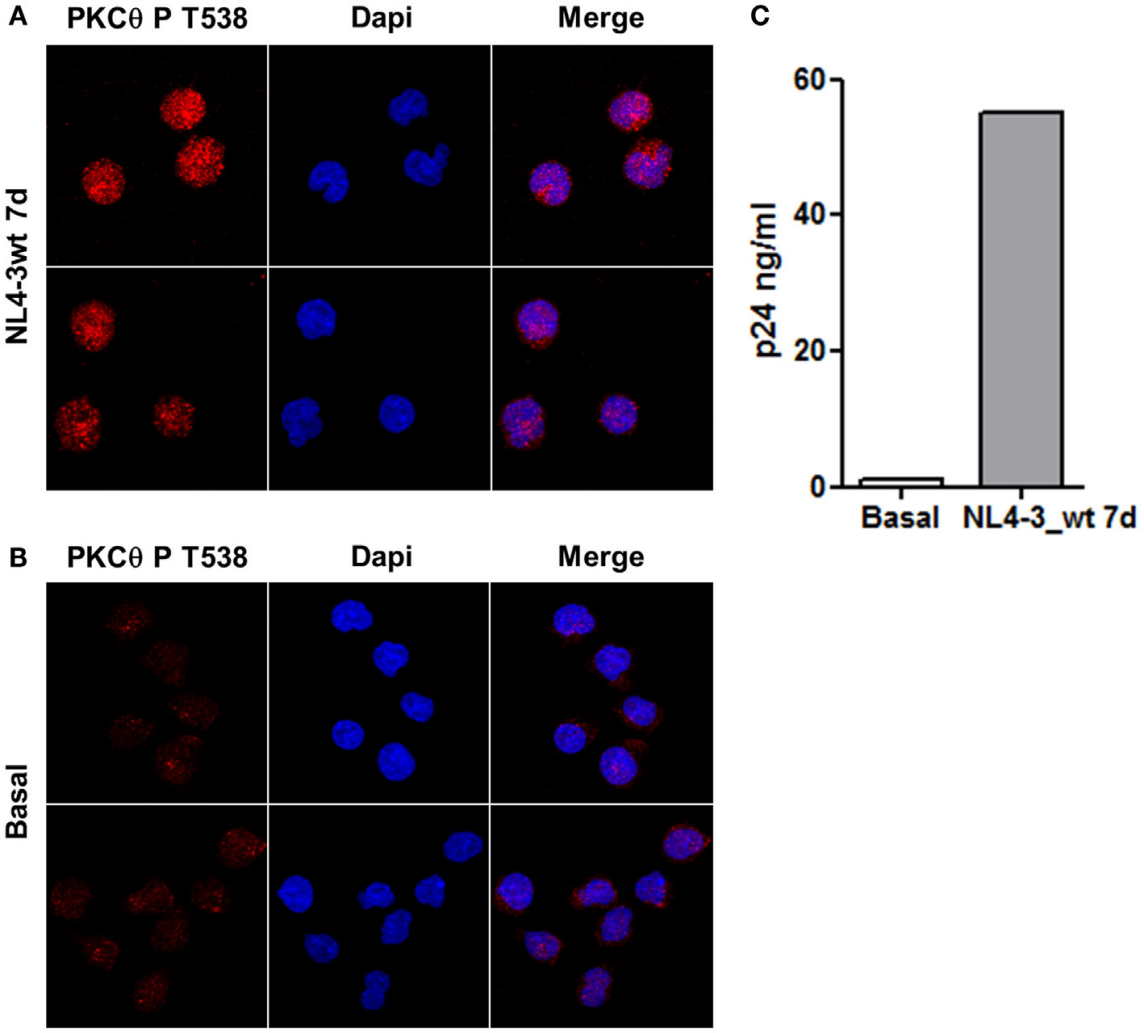
**Infection of Jurkat cells with HIV-1 induced PKCθ phosphorylation at T^538^**. Phosphorylation of PKCθ at T^538^ in Jurkat E6-1 infected with the infectious clone NL4-3_wt for 7 days **(A)** were analyzed by confocal microscopy and compared with uninfected cells **(B)**. Progression of the infection was determined by quantification of HIV-1 p24 (ng/ml) 7 days postinfection **(C)**.

Jurkat E6-1 cells infected with HIV-1 clone NL4-3_wt for 7 days were stained with an antibody against PKCθ phosphorylated at T^538^ and analyzed by confocal microscopy. Levels of PKCθ phosphorylated at T^538^ increased in infected cells (Figure 1A) compared to uninfected control cells (Figure 1B). Selection of the optical section through the middle of the cell – *z* axis – showed that phospho-PKCθ had an intracellular distribution, and it was also located inside the nucleus. Progression of the infection was monitored by quantification of p24 by ELISA after 7 days of infection (Figure 1C).

### PKCθ mRNA and Kinase Activity Are Enhanced in Jurkat Cells with Stable Expression of HIV-1 Tat

**Figure 2:**
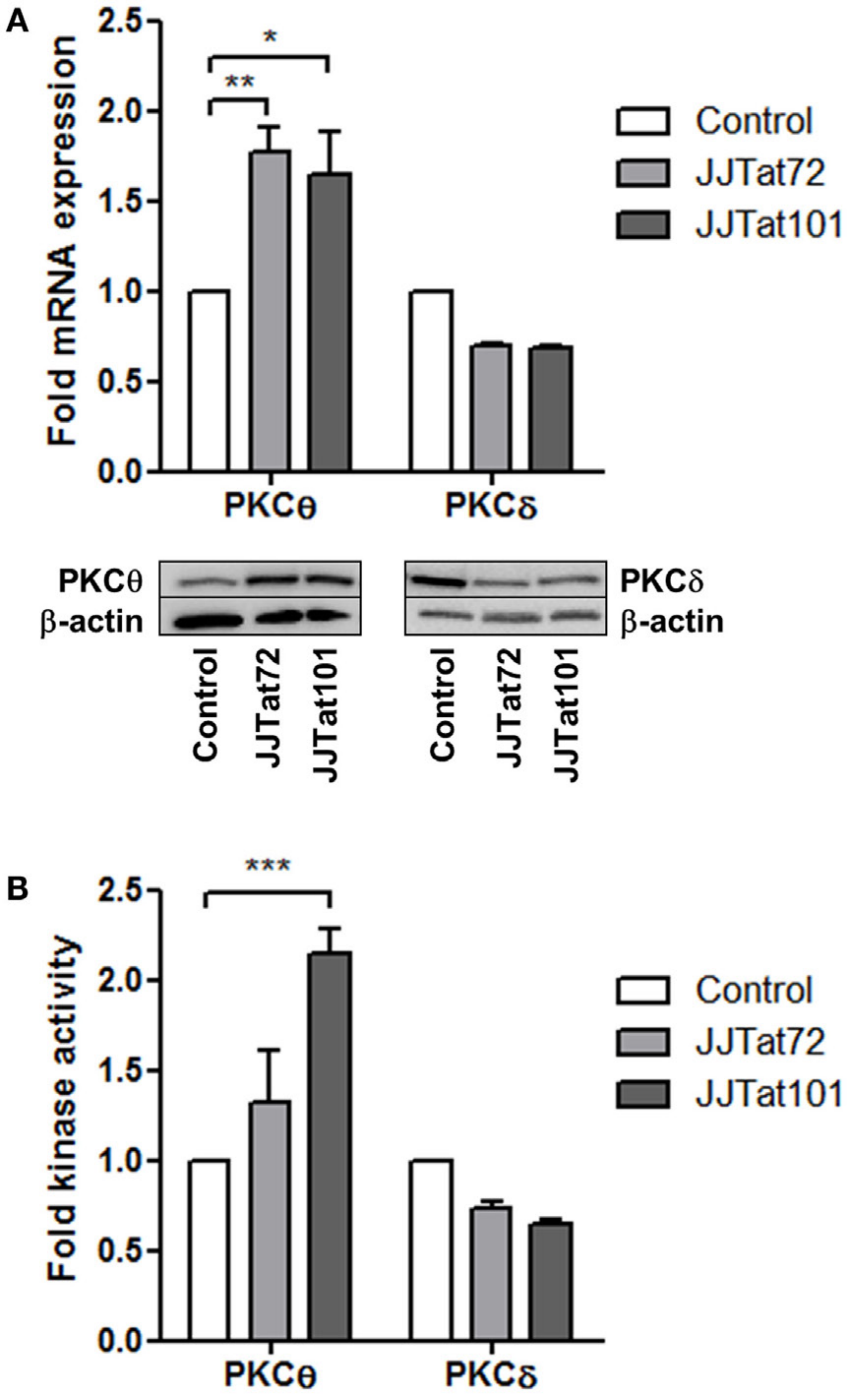
**PKCθ mRNA expression and kinase activity were enhanced in Jurkat-Tat101 cells**. Expression of mRNA and protein for PKCθ and PKCδ, analyzed by qPCR and immunoblotting, respectively, **(A)**, as well as kinase activity for PKCθ and PKCδ **(B)**, were quantified in Jurkat-Tat101 and Jurkat-Tat72, regarding control cells. Data are represented as mean ± SEM. *, **, or *** for *p* < 0.05, *p* < 0.01, or *p* < 0.001, respectively.

Expression of mRNA and protein for PKCθ and the structurally related PKCδ was analyzed by quantitative PCR (qPCR) and immunoblotting, respectively, in Jurkat cells with stable intracellular expression of Tat101 (codified by first and second exons) and Tat72 (codified by first exon). PKCθ expression level was increased in both Jurkat-Tat72 (*p* < 0.05) and Jurkat-Tat101 (*p* < 0.01) compared to control cells, whereas the expression of mRNA and protein for PKCδ was slightly reduced (Figure 2A). PKCθ and PKCδ kinase activities were analyzed to evaluate if the increase in mRNA was related to an increased kinase activity. PKCθ kinase activity was enhanced in Jurkat-Tat101 cells (*p* < 0.001) relative to control cells, and this increase was lower in Jurkat-Tat72 (Figure 2B). No significant change in PKCδ kinase activity was detected in Jurkat-Tat cells.

### Phosphorylation of PKCθ at T^538^ Was Increased in Jurkat-Tat Cells

**Figure 3:**
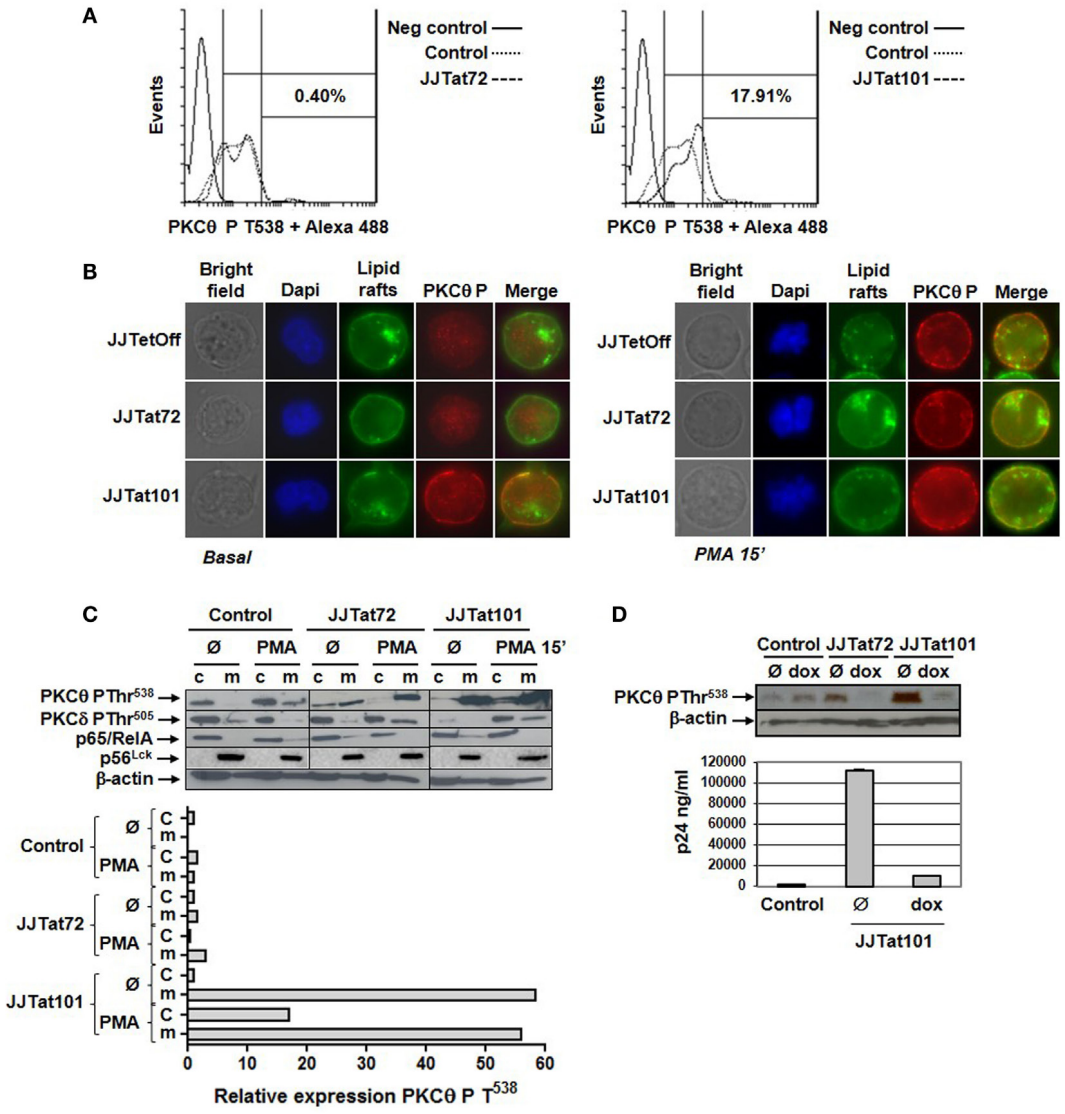
**Phosphorylation of PKCθ at T^538^ and translocation to the plasma membrane were increased in Jurkat-Tat101 cells**. Phosphorylation level of PKCθ at T^538^ was quantified in Jurkat-Tat cells by flow cytometry after intracellular staining, which was compared to the negative control and basal control (Jurkat TetOff) **(A)**. Phosphorylation of PKCθ at T^538^ and translocation to the plasma membrane were analyzed in Jurkat-Tat cells by fluorescence microscopy **(B)** and immunoblotting in basal conditions and after treatment with PMA for 15 min (c, cytosol; m, plasma membrane). Expression of p65 was used to detect contamination of plasma membrane proteins with cytosolic proteins and p56^Lck^ to detect contamination of cytosolic with plasma membrane proteins. Densitometry was performed to quantify the relative expression of phospho-PKCθ **(C)**. Levels of phospho-PKCθ were analyzed by immunoblotting in protein extracts from the plasma membrane of Jurkat-Tat cells transfected with pNL4-3_TatM1I and treated with doxycycline for 3 days. Progression of the infection was monitored by quantification of HIV-1 p24 (ng/ml) **(D)**.

Intracellular expression of phosphorylared PKCθ at T^538^ was analyzed by flow cytometry in Jurkat-Tat cells after staining with a specific antibody. Jurkat-Tat101 cells showed increased PKCθ phosphorylation (17.91% more than in control cells) in the absence of activation (Figure 3A, right histogram). There were no significant changes in Jurkat-Tat72 cells, in relation to control cells (Figure 3A, left histogram). Translocation of PKCθ phosphorylated at T^538^ was analyzed by immunofluorescence using a specific antibody. Lipids rafts were stained with FITC to identify the plasma membrane, and nuclei were stained with Dapi. In comparison to Jurkat-Tat72 and control cells, in non-dividing Jurkat-Tat101 cells, there was an increased localization of PKCθ phosphorylated at T^538^ on the plasma membrane (Figure 3B, left panel). Furthermore, treatment with PMA for 15 min induced translocation of phospho-PKCθ to the plasma membrane in dividing cells across all cell types (Figure 3B, right panel). This result was also analyzed by immunoblotting and relative quantification was performed by densitometry. In control cells, phospho-PKCθ at the cytosol was translocated to the plasma membrane within 15 min of PMA treatment (Figure 3C). In Jurkat-Tat72 cells, phospho-PKCθ was already detected at the plasma membrane in basal conditions, but it was increased after treatment with PMA. Interestingly, high quantity of phospho-PKCθ was already detected at the plasma membrane of Jurkat-Tat101 cells under basal conditions, a level that was comparable to the PMA treatment with no significant changes. Phosphorylation of PKCδ did not significantly change in both Jurkat-Tat cells in basal conditions but increased in the plasma membrane after treatment with PMA. Expression of p65/RelA and p56^Lck^ was used, respectively, to discard the possible contamination of plasma membrane proteins with cytosolic proteins or cytosolic proteins with plasma membrane proteins. β-actin was used as loading control.

The importance of increased PKCθ phosphorylation at T^538^ on Tat101 expression in Jurkat-Tat101 cells was further analyzed after treating these cells with doxycycline for 3 days to eliminate Tat101 expression using the TetOff system. Treatment with doxycycline completely impeded PKCθ phosphorylation at T^538^ in both Jurkat-Tat101 and Jurkat-Tat72 cells after transfection with pNL4-3_TatM1I clone, which remains non-infectious unless Tat is externally provided, as was determined by immunoblotting (Figure 3D). Progression of the infection was determined by quantification of HIV-1 p24 (ng/ml).

### Increased NF-κB Activity in Jurkat-Tat Cells Was Reduced by the Interference of mRNA for PKCθ

**Figure 4:**
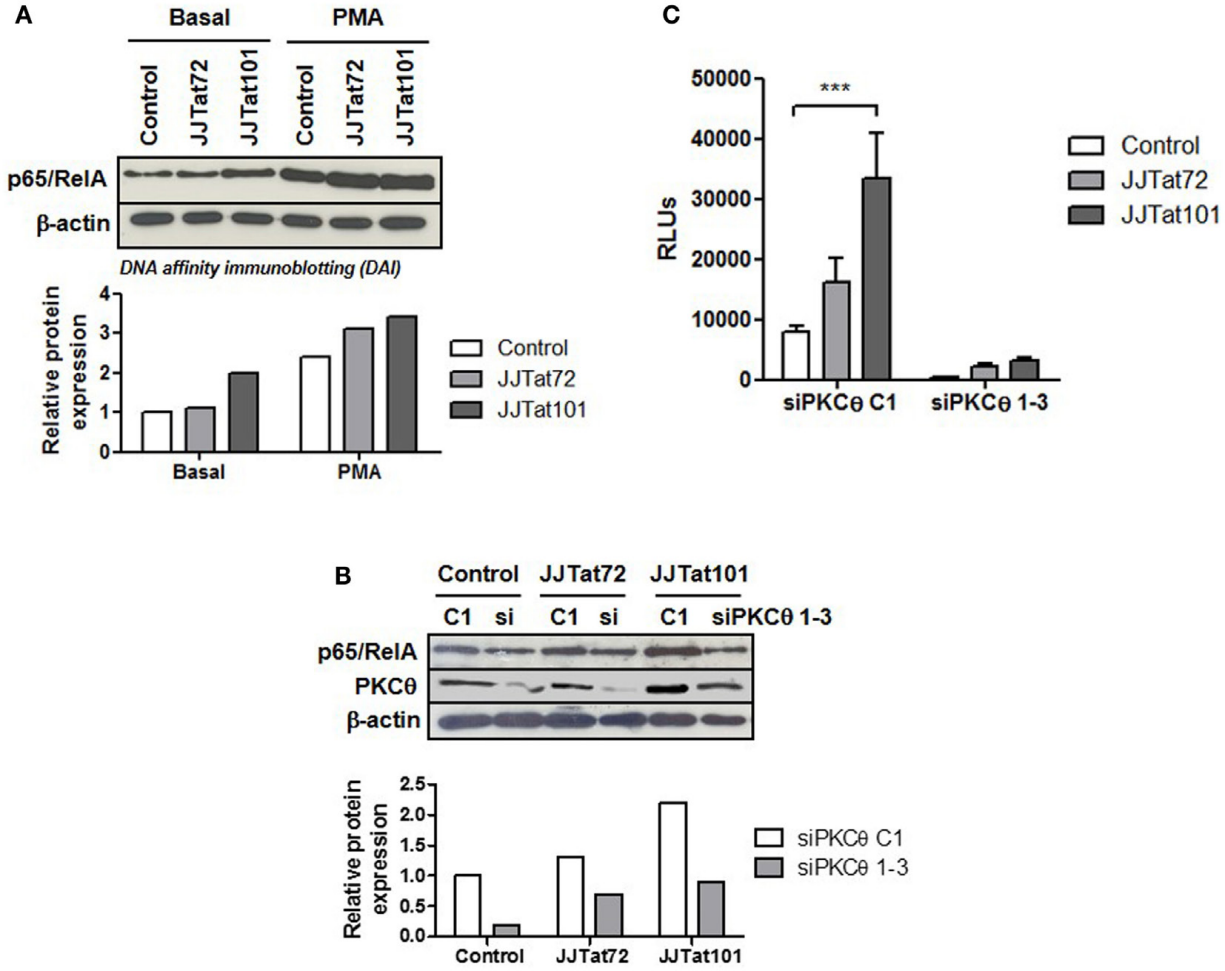
**Increased NF-κB activity in Jurkat-Tat cells was dependent on PKCθ activation**. Binding of p65/RelA to κB consensus sites in Jurkat-Tat cells treated or not with PMA was analyzed by DAI assay **(A)**. The same experiment was performed in Jurkat-Tat cells transfected with siRNA directed against mRNA for PKCθ. Analysis by immunoblotting of the level of PKCθ interference is shown **(B)**. Densitometry analysis was done to calculate relative p65/RelA expression and showed as horizontal bar diagrams **(A,B)**. Interference of mRNA for PKCθ in Jurkat-Tat cells transfected with a luciferase expression vector under the control of LTR promoter (pLTR-LUC) was analyzed by chemiluminescence. Data are represented as mean ± SEM. *** for *p* < 0.001 **(C)**.

One of the final effectors of PKCθ signaling pathway is NF-κB, a cellular transcription factor that is essential for T-cell survival (48) and HIV-1 replication (19). Regardless of PMA treatment, DAI demonstrated that there was an increased binding of p65/RelA – the most important component of NF-κB in T lymphocytes – to the κB promoter in Jurkat-Tat101 cells (Figure 4A). This increased NF-κB binding was inhibited in Jurkat-Tat101 cells by cotransfection of siRNA against PKCθ (Figure 4B), proving that increase in NF-κB activity was dependent on enhanced activation of PKCθ in Jurkat-Tat101 cells. Moreover, cotransfection of a Luciferase expression vector under the control of the LTR promoter (pLTR-LUC) and siRNA against PKCθ greatly reduced the production of LUC in Jurkat-Tat cells (Figure 4C). Transient transfection of shRNA plasmids pGeneClip-iPKCθ-1 and pGeneClip-iPKCθ-3 directed against mRNA encoding for PKCθ produced between 40 and 75% of stable RNA interference in Jurkat cells, as was previously described (20).

### Ras/Raf/MEK/ERK Pathway Was Increased in Jurkat-Tat Cells and Was Inhibited by RNA Interference for PKCθ

**Figure 5:**
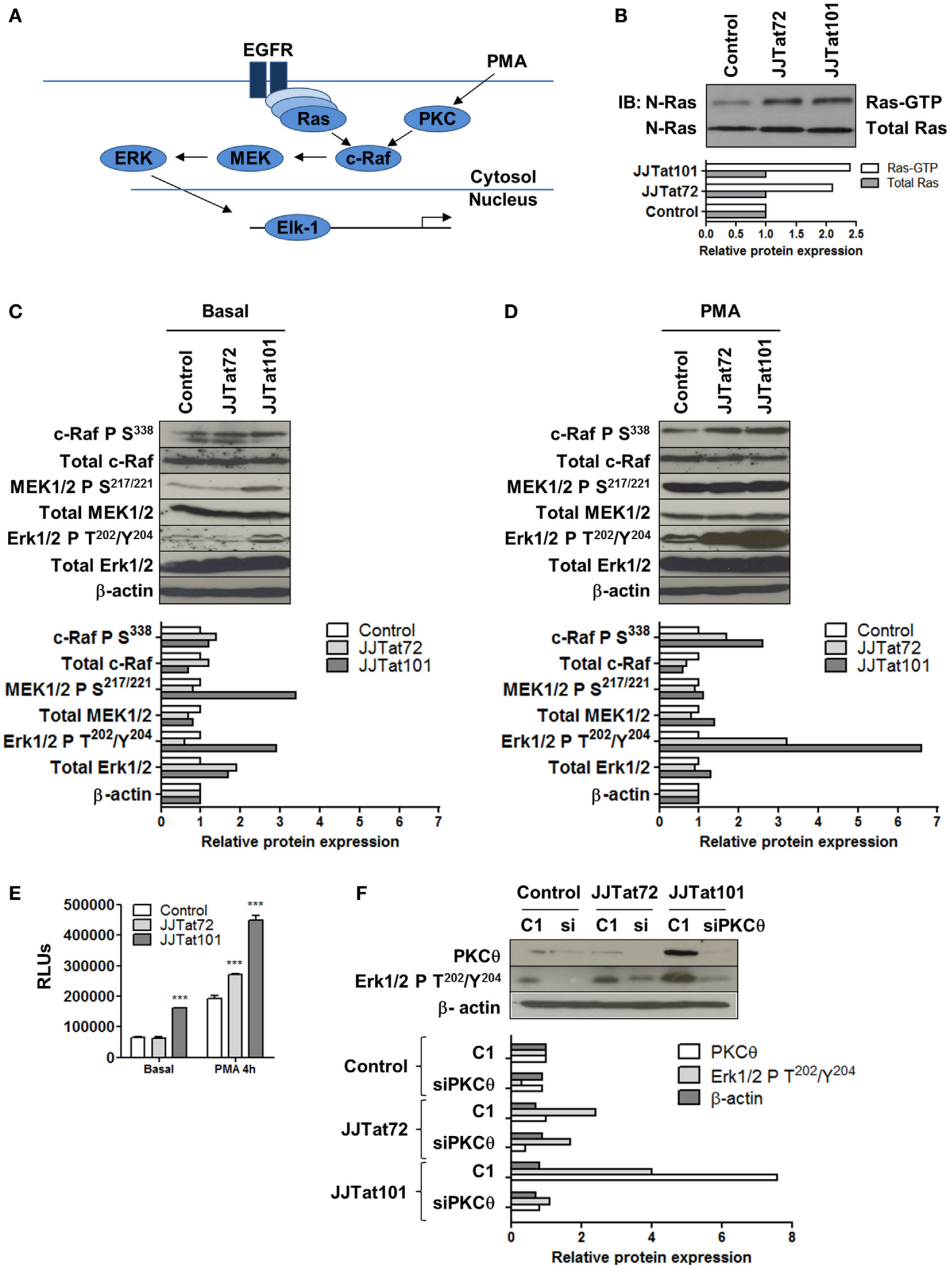
**Increased activation of Ras/Raf/MEK/ERK/Elk-1 pathway in Jurkat-Tat cells was dependent on PKCθ activation**. Schematic representation of the Ras/Raf/MEK/ERK/Elk-1 pathway (EGFR, epidermal growth factor receptor) **(A)**. Analysis by immunoblotting of Ras-GTP activity in Jurkat-Tat cells **(B)**. Analysis by immunoblotting of phosphorylated and total proteins of the Ras/Raf/MEK/ERK pathway in Jurkat-Tat cells in basal conditions **(C)** and after activation with PMA for 15 min **(D)**. Analysis by chemiluminescence of Elk1 activity in Jurkat-Tat cells in basal conditions and after treatment with PMA. Data are represented as mean ± SEM. *** for *p* < 0.001 **(E)**. Analysis by immunoblotting of levels of ERK1/2 phosphorylation at T^202^/Y^204^ after RNA interference of PKCθ in Jurkat-Tat cells **(F)**. Densitometry analyses were performed to calculate relative protein expression and showed as horizontal bar diagrams **(B–D,F)**.

Ras/Raf/MEK/ERK signal transduction pathway and the activation of the downstream transcription factor Elk-1 (Figure 5A) were analyzed in Jurkat-Tat cells to evaluate the role of PKCθ in activating this signaling pathway. First, Ras activity was analyzed in Jurkat-Tat cells by using Ras-GTP assay and it was increased in Jurkat-Tat cells regarding control cells (Figure 5B). Raf/MEK/Erk pathway was also analyzed by immunoblotting in Jurkat-Tat cells using specific antibodies against phosphorylation sites involved in the activation of c-Raf (S^338^), MEK1/2 (S^217/221^), and ERK1/2 (T^202^/Y^204^) (Figure 5C). All these sites were phosphorylated in Jurkat-Tat101 cells in basal conditions and phosphorylation further increased upon PMA treatment, mostly in ERK1/2 at T^202^/Y^204^ (Figure 5D). As a consequence, Elk-1 activity was also increased in Jurkat-Tat101 cells in basal conditions and after treatment with PMA for 4 h (Figure 5E). The role of PKCθ in this signaling pathway was determined by transfection of siRNA against mRNA for PKCθ in Jurkat-Tat cells and subsequent analysis of ERK1/2 phosphorylation at T^202^/Y^204^ (Figure 5F). RNA interference for PKCθ caused more than seven times reduction in ERK1/2 phosphorylation in Jurkat-Tat101 cells, compared to cells without interference.

### PKCθ Coexisted with Tat at the HIV-1 LTR Promoter in Jurkat Cells

**Figure 6:**
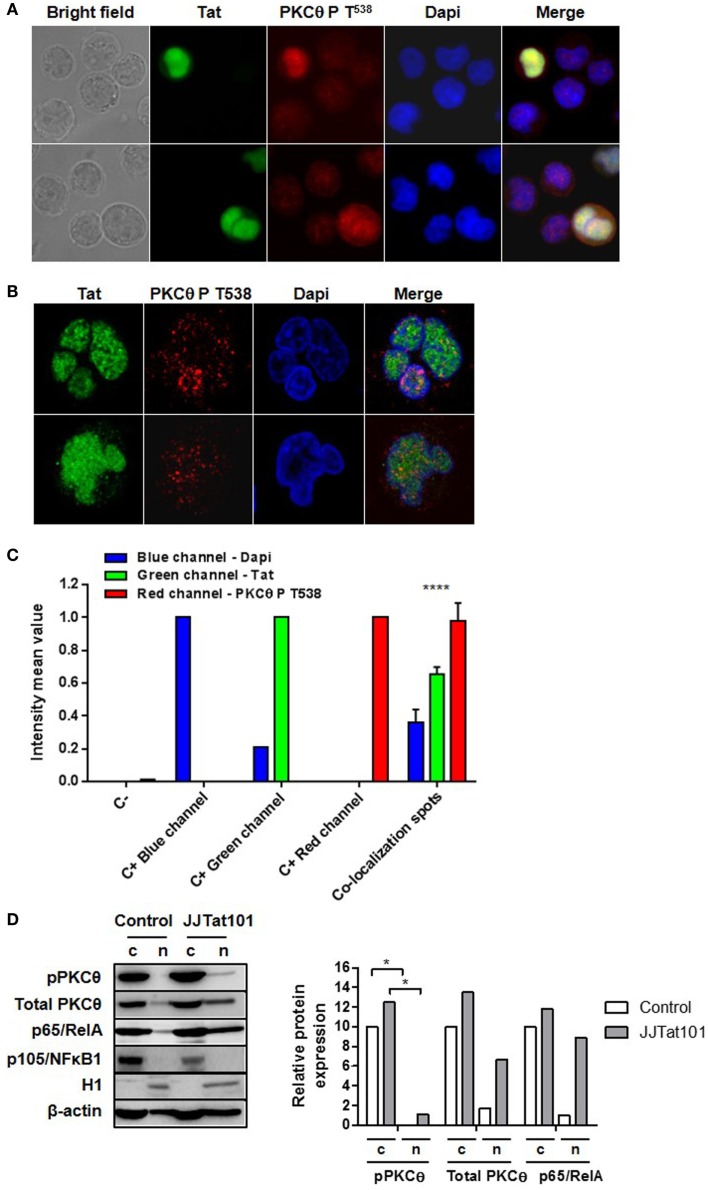
**Nuclear colocalization of PKCθ and Tat in Jurkat cells**. Nuclear colocalization of PKCθ and Tat was analyzed in Jurkat E6-1 cells transiently transfected with pCMV-Tat101 for 48 h by using fluorescence microscopy **(A)** and confocal microscopy **(B)**. The intensity mean per pixel was calculated in colocalization spots and values were represented in bar diagrams showing statistical significance **(C)**. Data are represented as mean ± SEM. **** for *p* < 0.0001. Nuclear presence of total and phosphorylated (T^538^) PKCθ was also analyzed by immunoblotting. Expression of p65/RelA was also determined. Analysis of p105/NFκB1 was used to detect contamination of nuclear proteins with cytosolic proteins and histone H1 to detect contamination of cytosolic (c) with nuclear (n) proteins. β-actin was used as internal loading control. Densitometry analyses were performed to calculate relative protein expression, according to β-actin for each lane, and showed as a bar diagram **(D)**.

Phosphorylation of PKCθ at T^538^ and its translocation to the plasma membrane is usually used as a surrogate marker of PKCθ activation (4–6, 14). However, we also detected this phosphorylated form of PKCθ in the nucleus by immunofluorescence of HIV-infected Jurkat cells (Figure 6A). Because increased PKCθ activity appeared to be dependent on the intracellular expression of Tat in Jurkat-Tat101 cells, we analyzed whether phospho-PKCθ was colocalizing with Tat in the nucleus. In order to synthesize large quantity of Tat, Jurkat E6-1 cells were transiently transfected with Tat101 expression vector (pCMV-Tat101) and incubated for 48 h. Analysis by immunofluorescence microscopy proved that those cells with higher expression of Tat101 showed also higher phosphorylation of PKCθ at T^538^ with a preferential nuclear distribution (Figure 6A). Analysis of these cells by confocal microscopy of an optical section through the middle of the cell – *z* axis – determined the nuclear localization of both Tat and PKCθ (Figure 6B). The intensity mean per pixel was calculated in colocalization spots using LAS AF Lite software (Leica Microsystems), and values were represented in bar diagrams showing statistical significance (Figure 6C). The presence of PKCθ in the nucleus was also analyzed by immunoblotting in Jurkat-Tat101 cells. PKCθ showed nuclear localization in Jurkat-Tat101 and part of it was phosphorylated at T^538^, in comparison with control cells (Figure 6D). This correlated with a higher nuclear localization of p65/RelA. Expression of p105/NFκB1 and the histone H1 was used to discard the possible contamination of nuclear proteins with cytosolic proteins or cytosolic proteins with nuclear proteins, respectively. β-actin was used as loading control and as control for the densitometry analysis.

**Figure 7:**
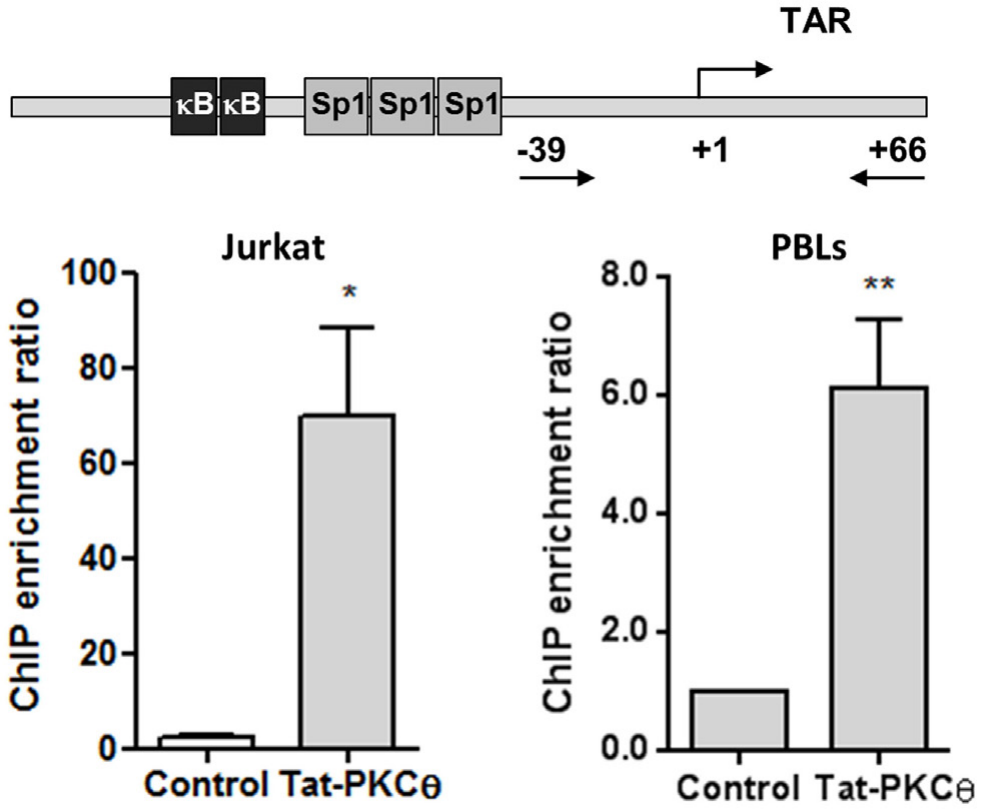
**Nuclear coexistence of PKCθ and Tat in Jurkat cells and human PBLs**. Nuclear proteins from Jurkat E6-1 cells transiently transfected with pCMV-Tat101 for 48 h or from PBLs infected with NL4-3_wt for 3 days were analyzed by sequential ChIP assay to detect the interaction between PKCθ and Tat in the HIV-1 LTR promoter, specifically in the region from −39 to +66 that contains the TAR loop (+1 to +59). ChIP enrichment ratio of two independent experiments is represented. Data are represented as mean ± SEM. * and ** for *p* < 0.05 and *p* < 0.01, respectively. Schematic representation of the proximal HIV-1 LTR promoter show the three binding sites for Sp1 and the two binding sites for NF-κB, located upstream TAR loop.

To evaluate whether Tat and PKCθ were directly interacting at the HIV-1 LTR promoter, sequential immunoprecipitation of chromatin (ChIP) was performed with DNA obtained from Jurkat E6-1 cells transfected with HIV-1 pNL4-3_wt infectious clone and from human PBLs infected with NL4-3_wt strain for 3 days. A primary ChIP was carried out using an antibody against Tat followed by a secondary ChIP using an antibody against PKCθ. qPCR analysis was performed on immunoprecipitated DNA using primers directed against specific regions of HIV-1 LTR promoter. Sequential ChIP analysis revealed that Tat and PKCθ coexisted on the HIV gene promoter in both Jurkat and PBLs (Figure 7), and that PKCθ was enriched at the region located within −39 and +66 that includes TAR loop. These sites could be the preferred binding sites for PKCθ at the HIV promoter.

## Discussion

### HIV-1 Infection Increased Phosphorylation of PKCθ at T^538^

HIV-1 primary infection is characterized by a massive activation of CD4^+^ T cells responsible for its depletion and for the generation of the viral reservoirs that impede the eradication of the infection (4–6). HIV-1 can infect resting and activated CD4^+^ T cells but full replication can only occur in activated cells (9). Therefore, control of CD4^+^ T cell activation during HIV-1 primary infection could regulate viral replication, decreasing the size of the reservoir, and reducing the depletion of CD4^+^ T cells. In this regard, our group described previously the important role of PKCθ in HIV-1 replication, as the interference of mRNA for PKCθ (20) or the use of selective PKCθ inhibitors (21) hindered HIV-1 replication in CD4^+^ T cells. We also observed that HIV-1 infection induced PKCθ activation by increasing its phosphorylation at T^538^ and subsequent translocation to the plasma membrane, events that are usually used as surrogate markers of PKCθ kinase activity (14). However, the mechanism by which HIV-1 could induce PKCθ phosphorylation in the absence of activating stimuli remains unknown.

### Intracellular Expression of Tat101 Increased PKCθ Activity in Jurkat Cells

Activation of PKCθ initiates several transducing pathways in T cells such as Ras/Raf/MEK/ERK and NF-κB (17). Both ERK and NF-κB have been described as essential for HIV-1 replication (19, 49, 50). They act in synergy with viral proteins as Nef (51) and the viral regulator Tat (19) to increase HIV-1 transcription. Tat is responsible for efficient transcription and elongation of the viral transcripts because it binds to TAR loop at the LTR promoter and recruits cellular factors such as P-TEFb to increase RNAPII hyperphosphorylation of the CTD (27). Fully active Tat protein (Tat101) is codified in two separated exons that conform a unique mRNA sequence after multiple splicing (52), but the protein codified by the first exon (Tat72) retains most of the ability to induce an efficient viral transcription (30). Both Tat101 and Tat72 show a predominant nuclear localization in the infected cells, although Tat may also be released to the extracellular medium and captured by neighboring cells, causing cytotoxicity (53). Although the action of signaling kinases was traditionally thought to occur predominantly in the cytoplasm, a new nuclear role for PKCθ was recently described as part of an active complex with RNAPII, attached to specific promoter regions (22). Based on this assertion, we evaluated the possible interaction between PKCθ and Tat in the nucleus of CD4^+^ T cells and their effect on HIV-1 replication. The role of Tat on the activation of PKCθ signaling pathways as Ras/Raf/MEK/ERK/Elk-1 and NF-κB were also analyzed.

Our group developed Jurkat cell lines with stable intracellular expression of Tat101 and Tat72 by using the TetOff system. Jurkat-Tat72 and Jurkat-Tat101 cell lines are not clones but mixed populations in which more than 50% of the cells express high amounts of intracellular Tat101 or Tat72 protein (32). Tat intracellular expression could be abrogated in Jurkat-Tat cells after incubation with doxycycline for 3 days. It was observed that not only the expression of mRNA for PKCθ was increased in Jurkat-Tat101 cells but PKCθ kinase activity was also enhanced in these cells. In Jurkat-Tat72 cells, expression of mRNA for PKCθ was increased, but it did not translated into an increased kinase activity, proving that the presence of the second exon in Tat101 was crucial for this effect of Tat on the activation of PKCθ. Phosphorylation of PKCθ at T^538^ was increased in Jurkat-Tat101 cells but not in Jurkat-Tat72, which correlated with the absence of enhanced kinase activity in these cells. Moreover, phospho-PKCθ was translocated to the plasma membrane in non-dividing Jurkat-Tat101 cells and in the absence of stimulation such as PMA treatment. Correlation with the intracellular expression of Tat101 in Jurkat-Tat101 cells and the increased PKCθ activity was demonstrated by treatment of Jurkat-Tat101 cells with doxycycline for 3 days, which completely inhibited PKCθ phosphorylation at T^538^ and its translocation to the plasma membrane, along with Tat functionality as was assessed by transfection with Tat-defective clone pNL4-3_TatM1I. Enhanced PKCθ activity in Jurkat-Tat101 appeared to be restricted to this kinase as the activity of the closely related novel PKCδ was not modified.

### Activation of Ras/Raf/MEK/ERK/Elk-1 and NF-κB Pathways in Jurkat-Tat101 Cells Was Dependent on Enhanced PKCθ Activity

We evaluated whether Ras/Raf/MEK/ERK/Elk-1 and NF-κB transduction pathways initiated by PKCθ were also activated in Jurkat-Tat101 in the absence of activating stimuli. Jurkat-Tat101 cells showed the main component of NF-κB in CD4^+^ T cells p65/RelA was bound to -κB consensus sequences. This binding was interfered by transfection of siRNA directed against mRNA for PKCθ, proving the link between Tat and PKCθ for the activation of NF-κB. Moreover, Jurkat-Tat101 showed high LTR-dependent transcription that was also abrogated by RNA interference of PKCθ, proving the essential synergy between PKCθ-stimulated NF-κB and Tat to initiate LTR-dependent transcription. Similarly, Ras activity was enhanced in Jurkat-Tat101 cells in basal conditions. Consequently, Raf/MEK/ERK pathway was also activated, as was demonstrated by the detection of activating phosphorylated forms of each protein. Analysis of the activity of transcription factor Elk-1, located at the end of Ras/Raf/MEK/ERK pathway, confirmed the increased activation of this via in Jurkat-Tat101 cells. Phosphorylation of essential proteins in this pathway was especially significant in the case of ERK1/2 at T^202^/Y^204^, which was greatly increased in Jurkat-Tat101 after stimulation with PMA. RNA interference of PKCθ also inhibited the greatest ERK1/2 phosphorylation in Jurkat-Tat101, proving that Tat-mediated increased PKCθ activity was responsible for this event.

### PKCθ and Tat101 Coexisted and Interacted in the Nucleus of Jurkat Cells, Specifically in the Region of the LTR Promoter that Contains TAR Loop

Once that a relationship between intracellular Tat expression and increased PKCθ activity was demonstrated, we tried to determine whether both proteins could coexist and interact in the nucleus of CD4^+^ T cells. It was observed by fluorescence microscopy that transient overexpression of Tat101 in Jurkat cells greatly increased PKCθ phosphorylation at T^538^ in the nucleus. Nuclear colocalization of both proteins was determined in an optical section through the middle of the cell – *z* axis – by confocal microscopy. Calculation of intensity mean values per each channel determined the presence of colocalization spots inside the nucleus. The possible interaction between Tat and PKCθ was confirmed by sequential ChIP in nuclear protein extracts from Jurkat cells transfected with the HIV-1 infectious clone pNL4-3_wt and from PBLs infected with NL4-3_wt for 3 days. ChIP results showed that Tat and PKCθ were interacting at the downstream region of the HIV-1 LTR promoter, specifically in the region that contains TAR loop, where Tat binds to ensure an efficient elongation of the viral transcripts. Intriguingly, transient overexpression of Tat101 or *in vitro* acute HIV-1 infection induced a high level of phosphorylation of PKCθ at T^538^ and its preferential translocation to the nucleus of the infected cell. However, stable expression of Tat101 in an established cell line caused translocation of phospho-PKCθ mostly to the plasma membrane, where PKCθ would activate several transduction pathways but not directly interact with Tat in the nucleus, contributing indirectly to HIV-1 replication through the activation of essential factors such as ERK and NF-κB. Both mechanisms could be related to the different viral replication level observed *in vivo* during acute and chronic infection.

In conclusion, in this study, we described the effect of stable expression of intracellular Tat101 and Tat72 on the activity of the essential kinase PKCθ in CD4^+^ T cells. Intracellular Tat101 was more potent at enhancing PKCθ activity that Tat72, proving that although Tat72 may be sufficient for the elongation of viral transcripts, the presence of the second exon in Tat101 greatly increased its positive effect on PKCθ kinase activity. Tat101 was able to induce PKCθ phosphorylation at T^538^ and its translocation to the plasma membrane or to the nucleus, in the absence of activating stimuli, promoting the activation of signal transduction pathways such as Ras/Raf/MEK/ERK and NF-κB, essential for HIV-1 full replication. Due to the importance of PKCθ for T-cell activation, more analysis will be needed to confirm the role of nuclear PKCθ to directly enhance viral transcription during HIV-1 infection and not only indirectly through the activation of transcription factors such as NF-κB and Elk-1. Nuclear coexistence between PKCθ and Tat during acute HIV-1 infection could be used as a therapeutic target because selective PKCθ inhibitors could disrupt this association and avoid the massive HIV-1 replication and CD4 depletion that occur during the first stages of the illness.

## Author Contributions

MC and JA conceived the study and interpreted the results. ML-H performed the immunofluorescence assays and most immunoblotting assays. SR-M and EM performed transfections, kinase activity, flow cytometry, and qPCR assays. CG-D performed the study about Ras/Raf/MEK/ERK/Elk-1 pathway. JL, AZ, and SR performed the ChIP assays. All authors revised critically the contents of the article.

## Conflict of Interest Statement

The authors declare that the research was conducted in the absence of any commercial or financial relationships that could be construed as a potential conflict of interest.
